# Supplementary material for: Nanoscale patterning of collagens in C. elegans apical extracellular matrix
Source: Nat Commun. 2023 Nov 18;14:7506. doi: 10.1038/s41467-023-43058-9 (PMC10657453; doi:10.1038/s41467-023-43058-9)
Supplement: Supplementary file 1 — Supplementary Information [file 41467_2023_43058_MOESM1_ESM.pdf]

Adams, Pooranachithra et al. Supplementary Information

Supplementary Figures 1-5

Supplementary Table 1

Supplementary Videos 1-2

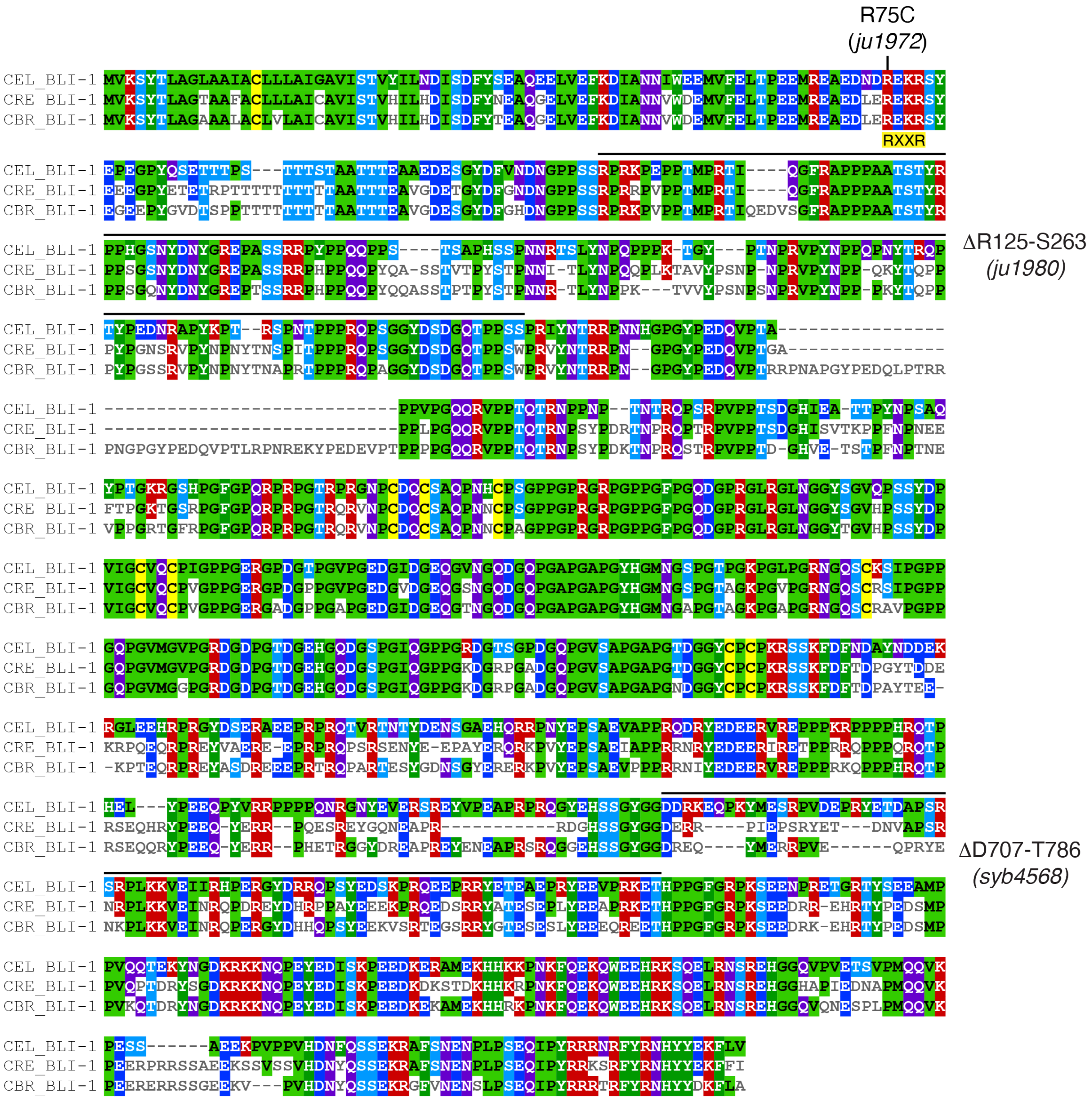

Supplementary Figure 1. BLI-1 orthologs in other Caenorhabditid species. Sequence alignment of BLI-1 orthologs in *C. remanei* (E3LFK7) and *C. briggsae* (A8WSY9). BLI-1 orthologs were identified using blast.caenorhabditis.org based on (1) overall sequence similarity, (2) presence of a central Gly-X-Y repeat domain and extended N- and C-termini, (3) similarities in the Cys motifs and other regions of similarity in the N-terminus. Sequences were aligned with Clustal Omega and displayed with MView [1]. *C. elegans* cuticle collagens have been classified based on features such as the pattern of Cys residues [2]; in this classification, BLI-1 is a member of group 1, BLI-2 of group 2, and BLI-6 of group 3. A more recent classification based on interruptions to

the Gly-X-Y repeats [3] placed BLI-1 in group C1, BLI-2 in group B6 and BLI-6 in group B14. The three *C. elegans* BLI collagens lack secretion signal sequences as predicted by SignalP 6.0 [4], but are predicted to have N-terminal transmembrane domains using DeepTMHMM [5]. The RXXR cleavage site is indicated in yellow. Residues predicted to be deleted by the in-frame deletions *ju1980* and *syb4568* are indicated.

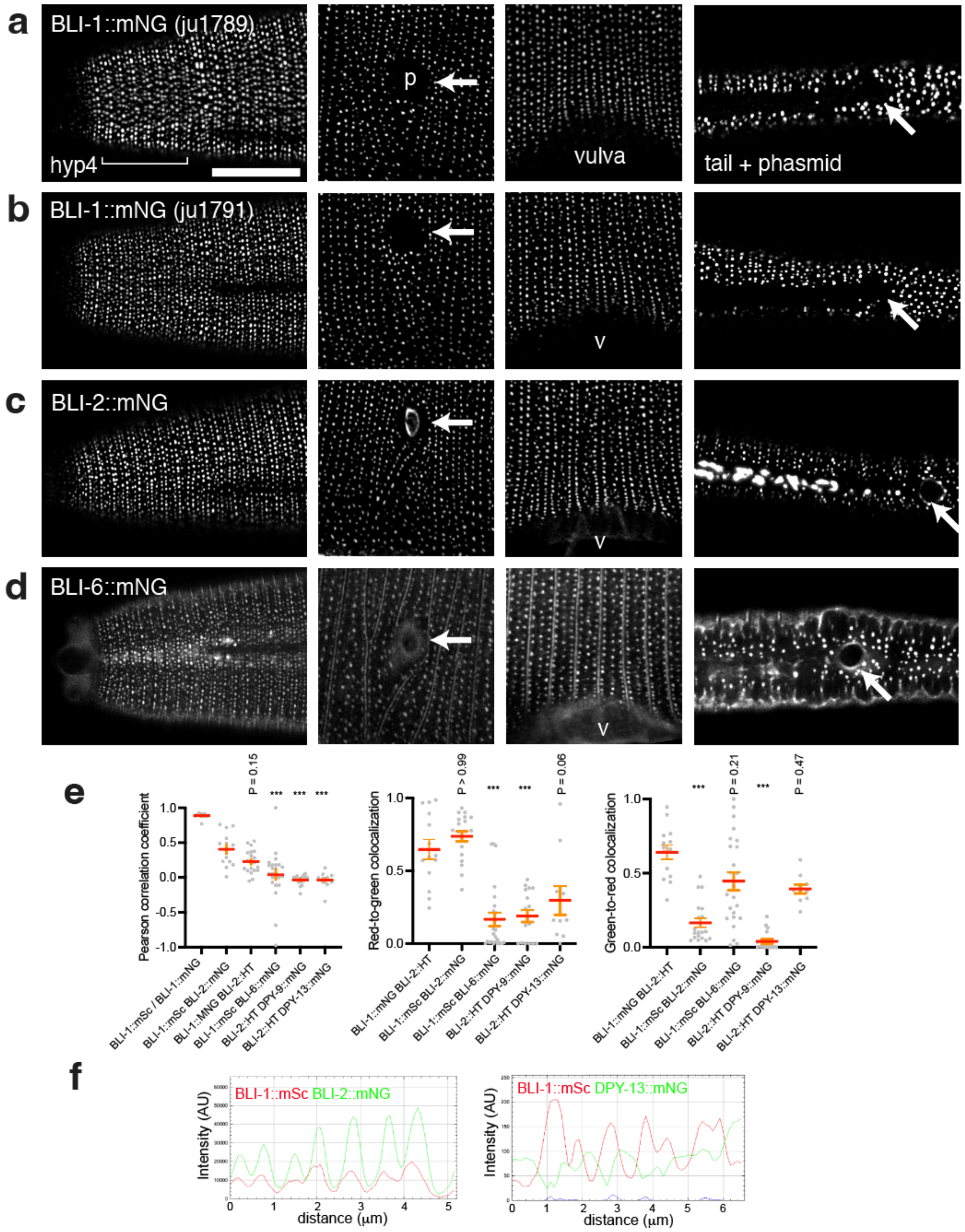

Supplementary Figure 2. Comparison of BLI localization and quantitation of BLI colocalization.

(a) BLI-1::mNG(*ju1789)* localization in anterior epidermis, postdeirid, vulva, and tail. BLI-1::mNG localizes to the cuticle overlying the anteriormost epidermal syncytia hyp3-5. BLI-1::mNG is excluded from the cuticle surrounding sensilla such as postdeirid and phasmid (arrows) and is not observed in vulval cuticle. BLI-1:;mNG puncta in the posteriormost cuticle (overlying hyp10) appear brighter than in the midbody. Lateral views, Zeiss LSM900 Airyscan imaging. Scale, 10 μm. (b) C-terminally tagged BLI-1 (*ju1791*) localization is similar to that of *ju1789*. (c) Localization of BLI-2::mNG is similar to that of BLI-1::mNG in the anterior cuticle. Unlike BLI-1::mNG, BLI-2::mNG is seen in ringlike structures surrounding the postdeirid and phasmid sensilla (arrows) and is faintly observed in the vulva cuticle. (d) BLI-6::mNG shows diffuse localization at the nose cuticle, in ringlike structures surrounding postdeirid and phasmid sensilla, and is strongly in the vulva cuticle. (e) Colocalization of BLI proteins. Pearson's correlation coefficients, red-green and green-red weighted colocalization coefficients. Data are from LSM800 confocal except BLI-1::mNG BLI-2::HT colocalization in 3D SIM; n = 3 imaging experiments per condition and approximately 3 ROIs per image. Data are shown as dot plots with mean and SEM. Statistics: Kruskal-Wallis test and Dunn's post test, *** P < 0.001. (f) Colocalization of BLI-1::mSc puncta. Line scans were drawn in confocal single slice images down the center of annuli and fluorescence intensity (arbitrary units) plotted using Plot Profile (Fiji). Peaks of BLI-1:;mNG align with peaks of BLI-2::mNG and with troughs in the DPY-13::mNG intensity.

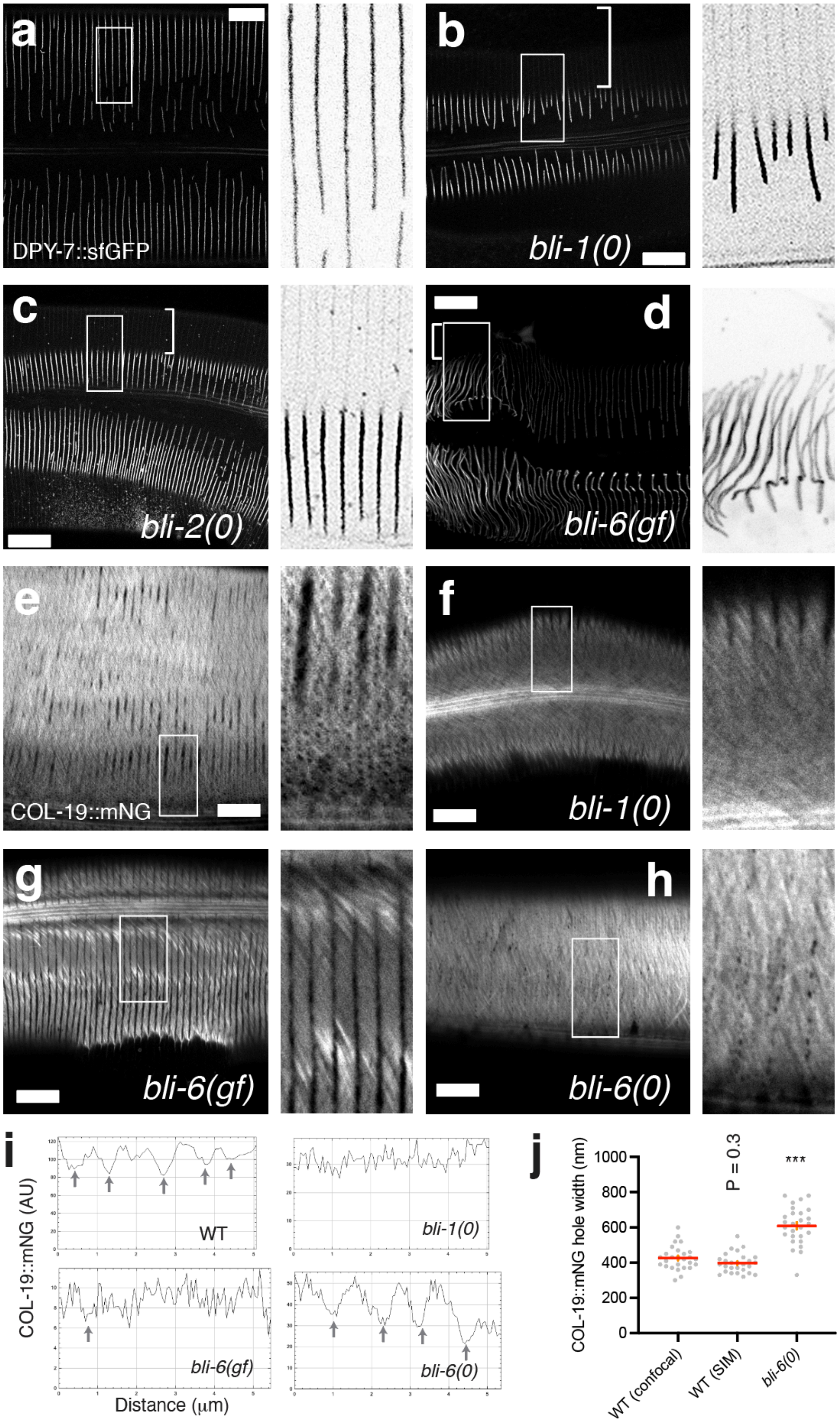
a
DPY-7::sfGFP
b
bli-1(0)
c
bli-2(0)
d
bli-6(gf)
e
COL-19::mNG
f
bli-1(0)
g
bli-6(gf)
h
bli-6(0)
i
COL-19::mNG (AU)
WT
bli-1(0)
bli-6(gf)
bli-6(0)
Distance (μm)
j
COL-19::mNG hole width (nm)
P = 0.3
***
WT (confocal)
WT (SIM)
bli-6(0)

Supplementary Figure 3. Furrow-Associated and Annulus-Associated collagen localization in *bli* mutants.

(a-d) DPY-7::sfGFP (*qxIs722*) localizes to regular circumferential bands corresponding to cuticle furrows in wild type. In *bli-1(0)* and *bli-2(0)* DPY-7::sfGFP localizes normally to furrows outside blistered areas and shows faint localization to furrows on basal and cortical sides of blisters; dorsoventral extent of blisters indicated by square bracket. DPY-7::sfGFP furrows are present in *bli-6(mn4)* but display aberrant organization. (e-h) COL-19::mNG(*syb4625*) localizes to fibrous bands underlying annuli, which are punctuated by regular small holes corresponding to struts. These holes are absent in *bli-1(0)* and *bli-6(gf)* animals and are present with abnormal patterning in *bli-6(0)*. Single focal planes in surface layer of COL-19::mNG. Scales, 10 μm; insets with inverted grayscale 10 μm wide. (i) Quantitation of COL-19::mNG holes in panels E-H. In images of single slices, line scans were drawn using Fiji 'Line Scan' along the center of annuli and gray values plotted using 'Plot Profile'. Holes, defined as regions > 0.5 μm of <20% peak intensity, are approximately 0.79 μm apart in the circumferential axis (n = 29 holes in 23 μm), consistent with strut circumferential spacing of 0.77 μm. The baseline of COL-19::mNG brightness (e.g. in *bli-1(0)*) varies due to the fibrous nature of COL-19 distribution. (j) COL-19::mNG hole sizes in WT (SIM and confocal) and in *bli-6(ju1681)*. n > 3 images, multiple line scans quantitated per image. Error bars are SEM. One-way ANOVA and Dunnett's post test, *** $P < 0.001$.

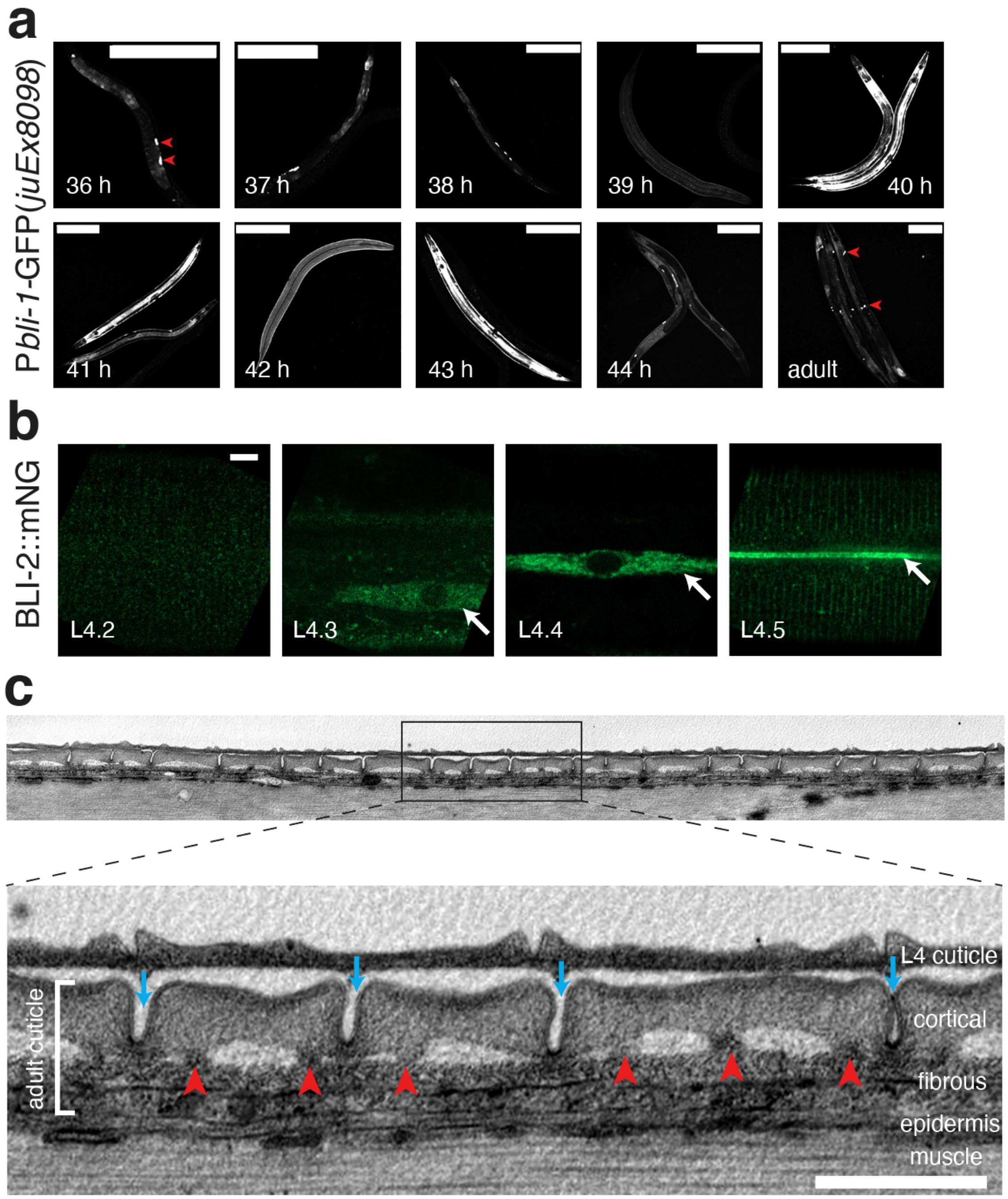

Supplementary Figure 4. *bli-1* transcriptional reporter and ultrastructure of the nascent adult cuticle in late L4 stage

(a) A *bli-1* transcriptional reporter (P*bli-1*-GFP, *juEx8098*) is upregulated in the epidermis in mid-L4 stage (L4.4 substage as defined by vulval morphology) and

downregulated in early adulthood. Times post L1 arrest at 20°C. The bright spots (arrowheads) visible at 36-38 h and in young adults are due to expression of the coinjection marker P*unc-122*-RFP in coelomocytes. GFP is seen throughout the hyp7 syncytium and excluded from the adult seam. Scales, 200 µm. (b). Diffuse localization of BLI-2::mNG in the lateral epidermis (seam cells, arrows at L4.3, L.4.4) and in alae at L4.5. Scale, 10 µm (c) Ultrastructure of developing adult cuticle in the L4; longitudinal section [6], L4.8/9 stage as defined by vulval morphology [7]. The L4 cuticle is outermost (top) and has partly separated from the developing adult cuticle. The nascent adult cuticle has formed cortical and fibrous layers; the furrows in the adult cuticle form deep indentations (blue arrows) whose bases are electron dense and closely apposed to the underlying fibrous layer. Two fibrous layers are visible with an electron dense interface. Nascent struts (red arrowheads) form adjacent to furrow bases or in the center of annuli and are 107 ± 5 nm in width (n= 37) at this stage; a medial layer is visible, but not a basal layer. Scale, 500 nm.

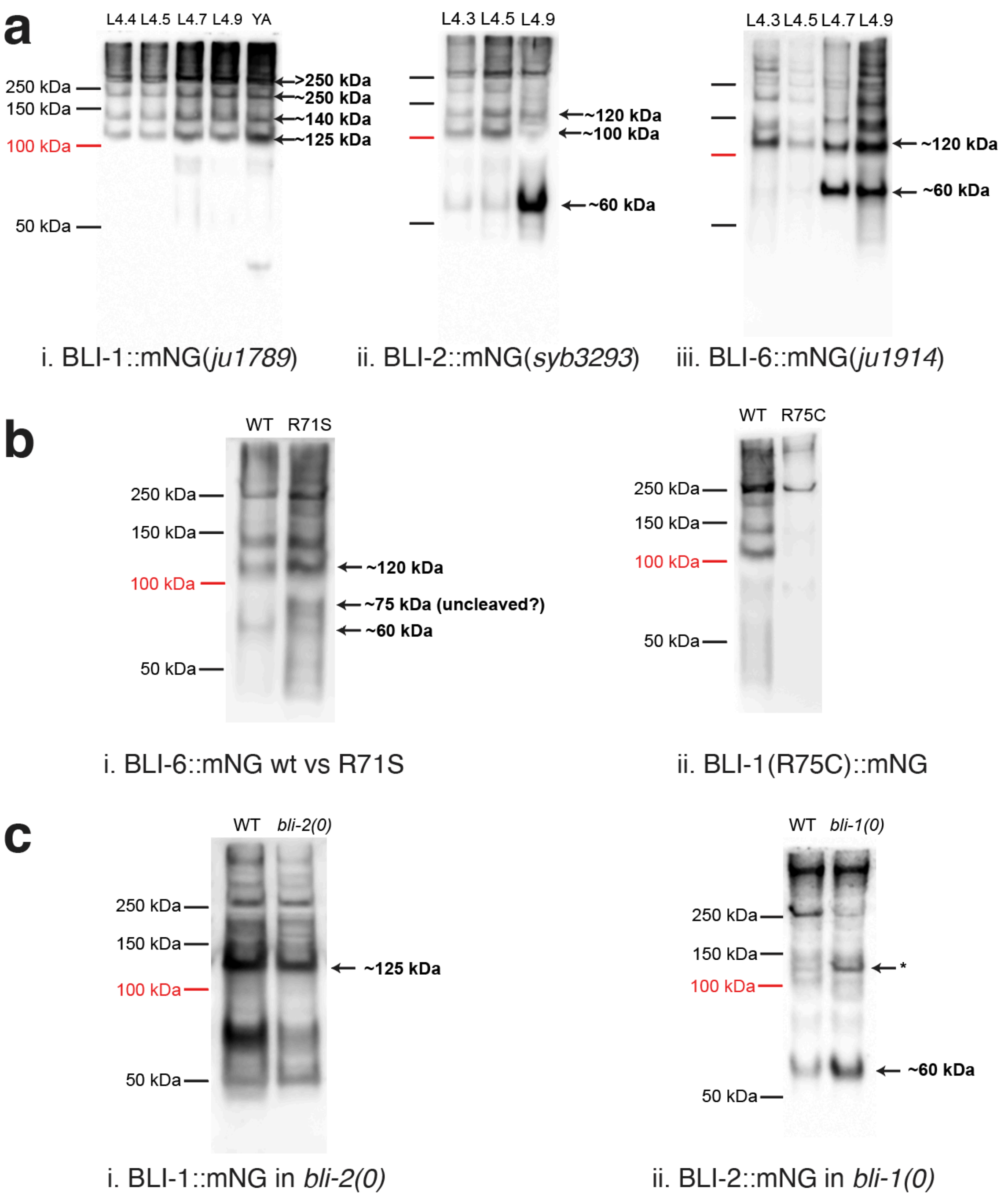

Supplementary Figure 5. **Biochemical analysis of BLI collagens during synthesis of adult cuticle in the L4 stage**

(a) Timecourse of mNG-tagged BLI proteins during L4 stage; western blots of soluble cuticle (F3) fractions. Size markers indicated at 250 kDa, 150 kDa, 100 kDa (red) and 50 kDa. (i) BLI-1::mNG was seen as a ~125 kDa band, consistent with monomeric BLI-

1::mNG after cleavage at the N-terminal cleavage site, starting in early L4, peaking in abundance at approximately L4.5 stage. Additional larger bands at ~250 kDa and > 250 kDa could correspond to dimeric and trimeric forms. Smaller bands of ~80 kDa and ~60 kDa were variably present in some preparations, possibly due to intracellular cleavage (see also panel B-ii). (ii) BLI-2::mNG appeared as multiple bands consistent with monomeric (~60 kDa), dimeric (~100-120 kDa) and higher order forms. (iii) BLI-6::mNG appeared as a single monomeric band (~60 kDa) and as ~120 kDa dimeric or higher order forms. (b) Effects of *bli* cleavage site mutations. (i) Extracts of BLI-6::mNG containing the cleavage site mutant R71S (*mn4*) displayed an additional band of ~75 kDa consistent with uncleaved BLI-6::mNG. (ii) The cleavage site mutant *bli-1(ju1972)* (R75C) introduced in the *ju1789* knock-in strain strongly reduced BLI-1::mNG accumulation in the soluble cuticle (F3) fraction. (c) Molecular epistasis of BLI proteins. (i) *bli-2(0)* mutants displayed reduced BLI-1::mNG accumulation in the soluble cuticle (F3) fraction. (ii) *bli-1(0)* mutants displayed reduced levels of higher molecular weight forms of BLI-2::mNG (>250 kDa) and increased levels of monomeric and dimeric forms. Extracts in panels B and C are from approximately L4.7 stage animals. Images are of representative blots (n = 3 per condition) and are not cropped beyond removal of the size marker lane and unloaded lanes.

Supplementary Table 1. Genetic interactions of *bli* mutants with other cuticle mutants

Mutation	Lesion or protein change	*bli-1(ju1395)*	*bli-2(ju1380)*	BLI-1::mNG
dpy-3(e182)	G177E	Sup (enhanced Dpy)	Sup	Disoriented rows
dpy-4(e1166)	1 bp insertion*	Sup	Sup	Normal rows
dpy-5(e61)	G203opal	Sup	Sup	Normal rows
dpy-7(e88)	G156R	Sup	Sup	Disoriented rows
dpy-8(e130)	G147R	Sup (< 5% Bli)	Sup	Disoriented rows
dpy-9(e12)	G149E	Sup	Sup	Disoriented rows
dpy-13(e184)	R75C and 30 bp deletion	Sup	Sup	Normal rows
dpy-17(e1295)	1 bp insertion*	100% Mild Bli, enhanced Dpy	74% Bli, enhanced Dpy	Some disorientation
lon-3(ok2076)	1472 bp deletion	100% Bli non-Lon	100% Bli non-Lon	Normal rows
sqt-2(sc3)	R75C	Sup	Partial Sup	Disrupted rows
*sqt-3(sc63*ts*)*	G227E	Partial Sup (84% Bli)	Partial Sup (38% Bli)	Some disorientation

Sup, suppressed to non-Bli (see Figure 6a); suppressed strains are 100% non-Bli unless stated (n = 50 per genotype). BLI-1::mNG patterns shown in Figure 6c.

* *e1166* causes a frameshift at codon 392 [8]. The *e1295* mutation is CCCC > CCCCC causing frameshift after codon P110. Mutations listed are thought to be partial loss of function or null apart from the *sqt* mutations which are thought to cause gain of function.

Supplementary Videos

Video 1. BLI-1::mNG (*ju1789*) timelapse imaging during L4 stage. Experiment 1298, 70 minutes imaged every 2 minutes. Inverted grayscale. Scale, 5 µm. See also Figure 4d.

Video 2. BLI-2::mNG (*syb3293*) timelapse imaging during L4 stage. Experiment 533, 116 min imaged every 4 minutes. Inverted grayscale. Scale, 5 µm.

References to Supplementary Information

1 Madeira, F. *et al.* Search and sequence analysis tools services from EMBL-EBI in 2022. *Nucleic Acids Res* (2022). https://doi.org:10.1093/nar/gkac240

2 Johnstone, I. L. Cuticle collagen genes. Expression in *Caenorhabditis elegans*. *Trends Genet* **16**, 21-27 (2000). https://doi.org:10.1016/s0168-9525(99)01857-0

3 Teuscher, A. C. *et al.* The in-silico characterization of the *Caenorhabditis elegans* matrisome and proposal of a novel collagen classification. *Matrix Biol Plus* **1**, 100001 (2019). https://doi.org:10.1016/j.mbplus.2018.11.001

4 Teufel, F. *et al.* SignalP 6.0 predicts all five types of signal peptides using protein language models. *Nat Biotechnol* **40**, 1023-1025 (2022). https://doi.org:10.1038/s41587-021-01156-3

5 Hallgren, J. *et al.* DeepTMHMM predicts alpha and beta transmembrane proteins using deep neural networks. *Biorxiv* (2022). https://doi.org:https://doi.org/10.1101/2022.04.08.487609

6 Cohen, J. D. *et al.* A multi-layered and dynamic apical extracellular matrix shapes the vulva lumen in *Caenorhabditis elegans*. *Elife* **9** (2020). https://doi.org:10.7554/eLife.57874

7 Mok, D. Z., Sternberg, P. W. & Inoue, T. Morphologically defined sub-stages of *C. elegans* vulval development in the fourth larval stage. *BMC Dev Biol* **15**, 26 (2015). https://doi.org:10.1186/s12861-015-0076-7

8 Simmer, F. *et al.* Genome-wide RNAi of *C. elegans* using the hypersensitive *rrf-3* strain reveals novel gene functions. *PLoS Biol* **1**, E12 (2003). https://doi.org:10.1371/journal.pbio.0000012
