# Supplementary material for: Nanoscale patterning of collagens in C. elegans apical extracellular matrix
Source: Nat Commun. 2023 Nov 18;14:7506. doi: 10.1038/s41467-023-43058-9 (PMC10657453; doi:10.1038/s41467-023-43058-9)
Supplement: Supplementary file 3 — Description of Additional Supplementary Files [file 41467_2023_43058_MOESM3_ESM.pdf]

Description of Additional Supplementary Files

File name: Supplementary Data 1
Description: Excel file of strains, primers, plasmids, and allele sequences.

File name: Supplementary Movie 1
Description: BLI-1::mNG (ju1789) timelapse imaging during L4 stage. Experiment 1298, 70 minutes imaged every 2 minutes. Inverted grayscale. Scale, 5 µm. See also Figure 4d.

File name: Supplementary Movie 2
Description: BLI-2::mNG (syb3293) timelapse imaging during L4 stage. Experiment 533, 116 min imaged every 4 minutes. Inverted grayscale. Scale, 5 µm.
