# Supplementary material for: Nanoscale patterning of collagens in C. elegans apical extracellular matrix
Source: Nat Commun. 2023 Nov 18;14:7506. doi: 10.1038/s41467-023-43058-9 (PMC10657453; doi:10.1038/s41467-023-43058-9)
Supplement: Supplementary file 7 — Reporting Summary [file 41467_2023_43058_MOESM7_ESM.pdf]

nature portfolio

Corresponding author(s): *Double-anonymous peer review submissions: write DAPR and your manuscript number here instead of author names.*

Last updated by author(s): *YYYY-MM-DD*

Reporting Summary

Nature Portfolio wishes to improve the reproducibility of the work that we publish. This form provides structure for consistency and transparency in reporting. For further information on Nature Portfolio policies, see our Editorial Policies and the Editorial Policy Checklist.

Statistics

For all statistical analyses, confirm that the following items are present in the figure legend, table legend, main text, or Methods section.

n/a	Confirmed	
☐	☒	The exact sample size (n) for each experimental group/condition, given as a discrete number and unit of measurement
☒	☐	A statement on whether measurements were taken from distinct samples or whether the same sample was measured repeatedly
☐	☒	The statistical test(s) used AND whether they are one- or two-sided<br>*Only common tests should be described solely by name; describe more complex techniques in the Methods section.*
☒	☐	A description of all covariates tested
☐	☒	A description of any assumptions or corrections, such as tests of normality and adjustment for multiple comparisons
☐	☒	A full description of the statistical parameters including central tendency (e.g. means) or other basic estimates (e.g. regression coefficient) AND variation (e.g. standard deviation) or associated estimates of uncertainty (e.g. confidence intervals)
☐	☒	For null hypothesis testing, the test statistic (e.g. F, t, r) with confidence intervals, effect sizes, degrees of freedom and P value noted<br>*Give P values as exact values whenever suitable.*
☒	☐	For Bayesian analysis, information on the choice of priors and Markov chain Monte Carlo settings
☒	☐	For hierarchical and complex designs, identification of the appropriate level for tests and full reporting of outcomes
☐	☒	Estimates of effect sizes (e.g. Cohen's d, Pearson's r), indicating how they were calculated

Our web collection on statistics for biologists contains articles on many of the points above.

Software and code

Policy information about availability of computer code

Data collection	Images were collected using commercial Zeiss microscopes using Zeiss ZEN software. 3D SIM data was acquired using a commercial Deltavision OMX microscope using Deltavision proprietary software Softworx.
Data analysis	Downstream image analysis or rendering used open-source Fiji (ImageJ v2.9.0) or Icy (https://icy.bioimageanalysis.org).

For manuscripts utilizing custom algorithms or software that are central to the research but not yet described in published literature, software must be made available to editors and reviewers. We strongly encourage code deposition in a community repository (e.g. GitHub). See the Nature Portfolio guidelines for submitting code & software for further information.

Data

Policy information about availability of data

All manuscripts must include a data availability statement. This statement should provide the following information, where applicable:

- Accession codes, unique identifiers, or web links for publicly available datasets
- A description of any restrictions on data availability
- For clinical datasets or third party data, please ensure that the statement adheres to our policy

Further information and requests for resources should be directed to and will be fulfilled by the lead contact, Andrew Chisholm (adchisholm@ucsd.edu). Numerical data for the quantitative analyses are provided with this paper (Source Data File 1). Image data files have been uploaded to Figshare at doi: 10.6084/m9.figshare.24353035

Research involving human participants, their data, or biological material

Policy information about studies with human participants or human data. See also policy information about sex, gender (identity/presentation), and sexual orientation and race, ethnicity and racism.

Reporting on sex and gender	Not applicable.
Reporting on race, ethnicity, or other socially relevant groupings	Not applicable.
Population characteristics	Not applicable.
Recruitment	Not applicable.
Ethics oversight	Not applicable.

Note that full information on the approval of the study protocol must also be provided in the manuscript.

Field-specific reporting

Please select the one below that is the best fit for your research. If you are not sure, read the appropriate sections before making your selection.

☒ Life sciences ☐ Behavioural & social sciences ☐ Ecological, evolutionary & environmental sciences

For a reference copy of the document with all sections, see nature.com/documents/nr-reporting-summary-flat.pdf

Life sciences study design

All studies must disclose on these points even when the disclosure is negative.

Sample size	Sample sizes for phenotypic analysis are based on standard practice in the C. elegans field. Sample sizes e.g. for puncta quantitation were determined by the size of the ROI and are in the range of several hundred data points per genotype, which is standard for puncta quantitation.
Data exclusions	No data were excluded.
Replication	Reproducibility of all C. elegans genetic data is ensured by use of multiple independent alleles of each gene and multiple knockin insertions. Imaging of each genotype was performed in at least 3 independent trials. Biochemical studies also used at least 3 independent trials and representative blot images are shown.
Randomization	Randomization is not used in C. elegans genetic experiments which are designed to compare specific genotypes.
Blinding	Blinding is not generally applicable as the samples (strains) being compared are clearly different in phenotype.

Reporting for specific materials, systems and methods

We require information from authors about some types of materials, experimental systems and methods used in many studies. Here, indicate whether each material, system or method listed is relevant to your study. If you are not sure if a list item applies to your research, read the appropriate section before selecting a response.

Materials & experimental systems

n/a	Involved in the study	
☐	☒	Antibodies
☒	☐	Eukaryotic cell lines
☒	☐	Palaeontology and archaeology
☐	☒	Animals and other organisms
☒	☐	Clinical data
☒	☐	Dual use research of concern
☒	☐	Plants

Methods

n/a	Involved in the study	
☒	☐	ChIP-seq
☒	☐	Flow cytometry
☒	☐	MRI-based neuroimaging

Antibodies

Antibodies used	Primary antibodies are all Anti-mNeonGreen (ChromoTek/ProteinTech clone No. 32F6). https://www.ptglab.com/products/mNeonGreenantibody-32F6.htm). Secondary antibodies are HRP-conjugated Goat anti-Mouse from Sigma, product # 12-349.

Validation	Validation of the primary mNeonGreen epitope-specific antibodies in our experimental setup is established by absence of signal in western blots of negative control (non-transgenic, N2) samples and by the correlation of expected band sizes with predicted band sizes in transgenic samples in Figure S5. Results are consistent across multiple lot numbers.

Animals and other research organisms

Policy information about studies involving animals; ARRIVE guidelines recommended for reporting animal research, and Sex and Gender in Research

Laboratory animals	C. elegans Bristol N2 is the reference wild type background for these studies. Specific strains are listed in our Supplementary Data file 1. Animals are aged between 40-80 h post hatching.
Wild animals	No wild animals were used.
Reporting on sex	C. elegans is a self reproducing hermaphrodite and animals studied are hermaphrodites unless specified. Sex was not considered as a variable except in Figure 3 reporting male specific patterns of collagen localization.
Field-collected samples	No field collected samples were used.
Ethics oversight	Ethical approval is not required for C. elegans studies under current US law.

Note that full information on the approval of the study protocol must also be provided in the manuscript.

Plants

Seed stocks	*Report on the source of all seed stocks or other plant material used. If applicable, state the seed stock centre and catalogue number. If plant specimens were collected from the field, describe the collection location, date and sampling procedures.*
Novel plant genotypes	*Describe the methods by which all novel plant genotypes were produced. This includes those generated by transgenic approaches, gene editing, chemical/radiation-based mutagenesis and hybridization. For transgenic lines, describe the transformation method, the number of independent lines analyzed and the generation upon which experiments were performed. For gene-edited lines, describe the editor used, the endogenous sequence targeted for editing, the targeting guide RNA sequence (if applicable) and how the editor was applied.*
Authentication	*Describe any authentication procedures for each seed stock used or novel genotype generated. Describe any experiments used to assess the effect of a mutation and, where applicable, how potential secondary effects (e.g. second site T-DNA insertions, mosiacism, off-target gene editing) were examined.*
